# *Tropheryma whipplei* native valve endocarditis diagnosed by sequencing of microbial cell-free DNA in plasma

**DOI:** 10.1128/asmcr.00070-25

**Published:** 2025-10-01

**Authors:** Peter M. Rabinowitz, Ritika Walia, Paul Pottinger, Joshua A. Lieberman

**Affiliations:** 1 Department of Environmental and Occupational Health Sciences, University of Washington 7284, Seattle, Washington, USA; 2 Department of Medicine, Division of Allergy and Infectious Disease, University of Washington 205280, Seattle, Washington, USA; 3 Department of Laboratory Medicine and Pathology, University of Washington 7284, Seattle, Washington, USA; Vanderbilt University Medical Center, Nashville, Tennessee, USA

**Keywords:** case report, *Tropheryma whipplei *endocarditis, next generation sequencing, metagenomics, cfDNA

## Abstract

**Background:**

Endocarditis is an important manifestation of extra-intestinal Whipple’s disease. The etiologic agent, the bacterium *Tropheryma whipplei*, cannot be cultivated in clinical laboratories, making the diagnosis of this culture-negative infection challenging. Molecular methods have emerged as useful adjuncts for the diagnosis of culture-negative endocarditis.

**Case Summary:**

A 67-year-old male was seen in an infectious disease clinic for evaluation of a possible infectious etiology of chronic musculoskeletal pain with exercise intolerance. He had a history of an embolic stroke 2 years earlier, echocardiographic evidence of aortic valve thickening, and multiple negative blood cultures. Following an evaluation that included serology and extended incubation blood culture, plasma was sent for metagenomic sequencing of microbial cell-free DNA, which was positive for *Tropheryma whipplei*.

**Conclusion:**

The patient’s musculoskeletal complaints and his exercise intolerance resolved after treatment with ceftriaxone and trimethoprim-sulfamethoxazole. To our knowledge, this is the first report of *T. whipplei* native valve endocarditis diagnosed by metagenomic sequencing.

## INTRODUCTION

*Tropheryma whipplei* is the causative agent for Whipple’s disease, a serious multisystem disorder and increasingly recognized as a cause of culture-negative endocarditis. The diagnosis of Whipple’s endocarditis has usually been made at the time of valve surgery through polymerase chain reaction (PCR) or histologic evaluation of tissue with histochemical stains, primarily Periodic acid–Schiff (PAS) and occasionally Warthin–Starry. We report a case of Whipple’s endocarditis diagnosed through metagenomic next-generation sequencing (mNGS) of microbial cell-free DNA (mcfDNA) from plasma. This case report follows the CARE guidelines (1).

## CASE PRESENTATION

A 67-year-old male presented to an infectious disease (ID) clinic with a complaint of persistent joint pain and concern for infection.

He reported being in good health until 6 years prior, when he noted the gradual onset of pain and stiffness in his right index finger and wrist, which over time developed into pain and stiffness in both hands and wrists (Table 1). X-rays of the left elbow and hand four years prior showed mild olecranon bursitis and degenerative changes at the base of the thumb. He continued to experience hand, arm, and wrist pain, and approximately 3 years prior also developed low back, hip, and buttock pain that worsened after movement. As a result, he gradually became unable to walk more than several hundred feet each day.

**TABLE 1:** Timeline of symptoms and key events

Timing^*a*^	Events and symptoms	Findings^*b*^
*t* −6 years	Onset of pain in wrists and hands	Mild inflammatory/degenerative changes on plain X-ray
*t* −3 years	Onset of back, hip, and buttock pain	N/A
*t* −2 years	Thrombotic stroke, blood cultures negative, long-term aspirin therapy	Negative blood cultures (four sets); negative coagulopathy testing
*t* −1 years	Prostatectomy, screening colonoscopy	High Gleason grade prostatic adenocarcinoma, confined to prostate; negative colonoscopy
*t* 0 years	Infectious disease (ID) clinic initial evaluation	Negative targeted serologies for *Bartonella* spp. and *Coxiella burnetii*; negative targeted PCR (blood) for *Brucella* spp.; negative extended hold blood cultures (×2)
*t* +0.5 years	Endocarditis treatment begun	TEE evidence of aortic valve involvement; plasma mNGS positive for *T. whipplei*
*t* +2 years	Asymptomatic, CRP and ESR within normal limits, antibiotics discontinued	N/A

^*a*^ − indicates years prior to evaluation in ID clinic; + indicates years after ID evaluation.

^*b*^ N/A indicates no additional findings.

Two years prior to his evaluation in the ID clinic, he experienced a middle cerebral artery ischemic stroke with left facial weakness and aphasia (see Timeline). He received alteplase and underwent successful mechanical thrombectomy with resolution of his neurological deficit. A transesophageal echocardiogram (TEE) found thickened aortic leaflets consistent with “non-bacterial thrombotic endocarditis.” Blood cultures from this outside hospital admission were collected from two peripheral sites over 3 days (six sets) and were negative at 5 days of growth. It is unclear from the records if anaerobic bottles were collected. Tests for antithrombin III, prothrombin variant, protein C and S, lupus anticoagulant, antiphospholipid, rheumatoid factor, ANA, and anti-CCP were within normal limits. A prostate-specific antigen was elevated. The patient was discharged on low-dose aspirin.

The patient had lived for the past 10 years on a 5-acre property in the outskirts of a small city in Washington State. He reported close contact with horses and livestock for more than 30 years. He reported frequently seeing wildlife such as rabbits and deer on his property.

The patient denied any recognized tick or mosquito bites. He had no recent travel outside of the USA. He reported rare consumption of alcohol and denied use of tobacco or other substances.

The patient’s past medical history was significant for prostate cancer (pT3aN0, Gleason 4 + 5) treated one year prior with a robotic-assisted laparoscopic prostatectomy and pelvic lymph node dissection. He had a history of hyperlipidemia. A colonoscopy 1 year before his visit to ID had been normal. His medication regimen included atorvastatin (80 mg daily), aspirin (81 mg daily), ibuprofen or aspirin as needed for pain, and daily chondroitin supplements.

At the time of evaluation by Infectious Diseases for his musculoskeletal complaints, the patient denied fever, night sweats, diarrhea, and weight loss. He also denied weakness, loss of sensation, or cognitive or speech difficulty. His exam was notable for trace bilateral pretibial edema, slight diffuse swelling and tenderness of his wrists and hands, and a BMI of 34. Initial lab testing revealed a mildly increased leukocyte count and a normal differential. Both ESR and C-reactive protein were increased. Prostate-specific antigen testing was within normal limits (Table 2).

**TABLE 2:** Laboratory findings upon initial outpatient evaluation by infectious diseases

Test	Result^*a*^	Reference range^*b*^
White blood cell count (WBC)	**10.93 × 10** ^**3**^ **/μL**	4.3–10.00 × 10³/μL
Polymorphonuclear cells	67%	N/A
Lymphocytes	22%	N/A
Monocytes	9%	N/A
Eosinophils	1%	N/A
Hematocrit	42%	38–50%
Hemoglobin	14 g/dL	13–18 g/dL
Platelets	313 × 10^3^/mL	150–400 × 10^3^/mL
Erythrocyte sedimentation rate	**18 mm/h**	0–15 mm/h
C-reactive protein	**14.8 mg/L**	0–10 mg/L
Prostate-specific antigen	<0.03 ng/mL	0–4.00 ng/mL

^*a*^ Values outside the reference range are in bold.

^*b*^ N/A indicates no applicable reference range.

Serologic studies for *Bartonella henselae*, *B. quintana*, *Brucella* spp., *Coxiella burnetii*, and *Chlamydia pneumoniae*, and *C. psittaci*, as well as *Bartonella* PCR of whole blood, were all negative. Qualitative *Francisella tularensis* serology was IgM negative and IgG positive. In consultation, the performing reference laboratory could not provide titers but described the result as “weakly positive” and possibly a biological false positive. Two extended incubation bacterial blood cultures (BACT/ALERT Virtuo), including an isolator tube, showed no growth at 2 weeks and 4 weeks, respectively. Five months following the initial evaluation in the ID outpatient clinic, a follow-up cardiology evaluation and a repeat TEE demonstrated moderate to severe thickening of the tips of all three aortic cusps.

After consultation with the laboratory medicine service, the decision was made to send the plasma for clinical mNGS of mcfDNA (Karius, Redwood, CA) (2). This testing detected *Trophyrema whippeli* at 314 DNA molecules per microliter (MPM) compared to the reference interval of <10 MPM. No other organisms were identified, and per the commercial laboratory’s report, this was the only *T. whipplei* detected in the last 1,000 specimens. Based on these results, the presence of the aortic valve vegetation, and the consistency of symptoms with published cases (3), a diagnosis of *Tropheryma* endocarditis was made. The patient received 4 weeks of intravenous ceftriaxone (2 g daily) and noted rapid resolution of his musculoskeletal pain and stiffness within days of starting treatment. After 4 weeks, therapy was transitioned to long-term twice-daily oral trimethoprim-sulfamethoxazole (800–160 mg, TMP-SMX). A transthoracic echocardiogram performed 18 months after initiation of therapy showed an improved left ventricular end-diastolic index (LEVDi) and reduced aortic regurgitation, consistent with healing of the vegetation. Two years after therapy began, he was able to discontinue TMP-SMX therapy and was reported that he had not needed to take ibuprofen or other pain medication since the beginning of the antibiotic treatment.

## DISCUSSION

This case illustrates not only the potential of NGS to diagnose unusual infections, such as Whipple’s endocarditis, but also the importance of detecting this particular condition. Whipple’s endocarditis is a disseminated form of Whipple’s disease caused by the bacterium *Tropheryma whipplei*. The epidemiology of the condition is poorly understood. A review of 169 published cases (3) reported a male sex predominance (85% male), with 52% of cases reporting primarily articular involvement and 41% presenting with heart failure. In that series, the majority of cases (156/169) were diagnosed directly on valve tissue: PCR (72%) and histology, including immunohistochemistry (51%) and/or PAS (39%) (3). The remaining seven cases had culture-negative valve vegetation in the setting of generalized Whipple’s disease (3). Treatment recommendations, based on small studies, involve an initial phase of treatment with ceftriaxone or penicillin, followed by a prolonged maintenance phase of treatment with TMP-SMX or alternatively doxycycline and hydroxychloroquine (3).

Authors have previously emphasized the importance of considering *T. whipplei* in the evaluation of culture-negative endocarditis (4). This case is a reminder that *T. whipplei* is also an important etiological consideration in the evaluation of patients with unexplained polyarthropathy (4, 5). In reported cases of Whipple’s endocarditis, arthralgia is a common complaint, and there is a risk of the underlying cause being missed. This risk is underscored by the report of a patient who underwent multiyear steroid treatment for arthritis before their *T. whipplei* IE was diagnosed, resulting in a fatally delayed diagnosis (6).

The timing of the patient’s ischemic CVA suggests that Whipple’s disease could have played a role in the cerebrovascular event. Previous studies have noted a high prevalence of ischemic stroke in patients with ischemic stroke and Whipple’s disease (7), and a recent systematic review found that embolic phenomena were reported in 28.2% of Whipple’s endocarditis cases (8).

While tissue diagnosis remains the gold standard for culture-negative endocarditis, the 2023 modified Duke criteria for infective endocarditis now recognize as a major criterion the molecular detection of *Coxiella burnetii*, *Bartonella* species, or *T. whipplei* DNA from blood (9). Molecular methods, both PCR and plasma mNGS, are particularly useful for non-cultivable organisms like *T. whipplei* and, if performed on peripheral blood samples, can provide a definitive diagnosis without invasive surgery. While mcfDNA has been reported to diagnose prosthetic valve endocarditis in the past and for other pathogens (10, 11), this is, to our knowledge, the first reported case of native valve Whipple’s endocarditis diagnosed by plasma mNGS. Although several studies have suggested mcfDNA concentrations fall in response to successful treatment (12, 13), no interpretive criteria currently exist to assess initial quantitation.

Although a promising method, plasma mNGS is expensive (~$2,200) and unlikely to be covered by insurance in the outpatient setting. Therefore, laboratories may employ intramural utilization guidelines to optimize ordering practices (14). Culture-negative endocarditis is a higher-yield use case for this assay (14), although false negatives (15) and false positives (16, 17) have been reported, and its utility depends on the clinical context, requiring careful test interpretation (16). Species-specific PCRs have been developed for *Coxiella* spp., *Bartonella* spp., and *T. whipplei*; when performed on blood, they are individually less expensive than plasma mNGS. However, costs quickly accumulate and collectively rival those of plasma mNGS, further emphasizing the importance of institutional ordering guidelines (14).

### Patient perspective

The patient had sought care because he was convinced that there was an infectious etiology for his chronic pain syndrome. At 1 year post-initiation of treatment, he reported that he felt “better and better all the time” and was able to walk several miles without difficulty.

### Conclusion

This case demonstrates the potential of non-invasive diagnostic techniques, such as sequencing of microbial cell-free DNA in plasma, to identify a causative agent in cases of culture-negative endocarditis.
